# A Method for Recording Urethral Pressure Profiles in Female Rats

**DOI:** 10.1371/journal.pone.0140851

**Published:** 2015-10-26

**Authors:** Shengfei Xu, Xiaohui Li, Lei Xu, Biao Chen, Huibing Tan, Guanghui Du

**Affiliations:** 1 Department of Urology, Tongji Hospital, Tongji Medical College, Huazhong University of Science and Technology, Wuhan, Hubei Province, P. R. China; 2 Department of Anatomy, Liaoning Medical College, Jinzhou, Liaoning Province, P. R. China; Max-Delbrück Center for Molecular Medicine (MDC), GERMANY

## Abstract

**Aims:**

Urethral pressure profile (UPP) and leak-point pressure (LPP) measurements as well as external urethral sphincter (EUS) electromyography (EMG) and videourodynamic analyses are the primary methods for evaluating urethral function in humans. However, UPP recording in female rats, a widely used animal model, is challenging due to their small body sizes. This study reports a novel method for recording UPP in female rats.

**Materials and Methods:**

Seventeen anesthetized female rats were studied. LPP data for 14 rats were included. The other 3 rats were excluded because of death or abnormal urogenital organs. UPP curves were recorded using a modified water-perfusion catheter system, with the lateral hole facing the 3-, 6-, 9-, and 12-o’clock positions in a randomized sequence. LPP, functional urethral length (FUL) and maximum urethral closure pressure (MUCP) were analyzed.

**Results:**

The mean LPP was 64.39 ± 20.29 cm H_2_O. The mean FUL and MUCP values at the 3-, 6-, 9-, and 12-o’clock positions were 12.90 ± 1.20, 16.70 ± 1.95, 13.90 ± 2.42, and 11.60 ± 0.97 mm, respectively, and 38.70 ± 11.85, 33.90 ± 11.82, 37.40 ± 11.95, and 71.90 ± 23.01 cm H_2_O, respectively. The FUL at the 6-o’clock position and MUCP at the 12-o’clock position were significantly greater than those at the other 3 positions. The FUL and MUCP of repeated UPP recordings were not significantly different than those of the first recordings.

**Conclusions:**

UPP recording using a modified method based on a water-perfusion catheter system is feasible and replicable in female rats. It produces UPP curves that sensitively and appreciably reflect detailed pressure changes at different points within the urethra and thus provides opportunity to evaluate urethral structures, especially the urethral sphincter, in detail. These results may enhance the utility of female rat models in research of urinary sphincter mechanisms.

## Introduction

Due to their ready availability, female rats are widely used for investigations of lower urinary tract functions and the pathophysiology of certain common clinical entities, such as urinary incontinence, urinary sphincter injury, and bladder outlet obstruction, as well as for the development of new therapeutic modalities [1–6]. Urethral pressure profile (UPP) and leak-point pressure (LPP) assessments as well as external urethral sphincter (EUS) electromyography (EMG), and videourodynamic analyses are the primary methods for evaluating urethral function in humans [7–12]. However, while LPP measurement via suprapubic catheterization and EUS-EMG with surgically implanted electrodes are well-established and generally accepted methods for use in rats, recording UPP in this animal is challenging due to its small body size [1–6,10–11]. To the best of our knowledge, only one previous study has record UPP curves in female rats, and it was conducted with a 1.4 Fr. Mikro-Tip catheter pressure transducer by Walters and colleagues in 2006 [13]. The latest research progress was made by a German group, who used a novel micro-tip catheter to record UPP in female minipigs [14].

UPP recording is a classic technique used in basic science and clinical practice for determining urinary sphincter dysfunction as a source of genuine stress incontinence or urethral obstruction [7,15–19]. Brown and Wickham were the first to describe and report a method for recording a constant UPP curve using a water-perfusion catheter system [20]. This technique provides measurements of pressure at consecutive points along the entire length of the urethra, and the sizes of these points are equal to those of the side holes in the catheter. This method has undergone many modifications and thorough investigations to standardize the water-perfusion rate, withdrawal speed, catheter diameter, and number and sizes of the catheter side holes [21]. These standards can be applied in humans and larger animal models; however, as stated above, rat UPP recording presents considerable technical difficulties because of the animal’s small body size. Small urethral diameters do not allow for the introduction of transurethral catheters with large diameters. Therefore, the fluid-filled balloon catheter and multiple-channel catheter that are typically used in human urodynamic studies are not suitable for rat UPP recording. The rat bladder volume is also small. The direct use of Brown and Wickham’s technique generally results in overextension of the bladder or an overflow of bladder urine, which interferes with the accurate measurement of urethral pressure. Therefore, UPP recording methods that are commonly used in humans are not directly suitable for use in rats.

We modified the technique described by Brown and Wickham in our female rat UPP recordings to allow for the recording of UPP curves that mimics those for humans. We used a 3 Fr. single-channel catheter with one side hole for the measurement of urethral pressure, which allowed for easy transurethral catheterization and good pressure transmission. The suprapubic bladder catheter remained open to allow fluid from the water-perfusion catheter to flow freely from the bladder during the UPP recordings and to overcome the limitation of the small bladder volume. UPP curves that sensitively and appreciably reflected detailed pressure changes at different points within the urethra were successfully recorded in female rats using these modifications.

## Materials and Methods

### Ethics Statement

The experimental designs and all procedures were performed in accordance with the National Institutes of Health Guide for the Care and Use of Laboratory Animals. The ethics committee of the Tongji Hospital, Tongji Medical College, Huazhong University of Science and Technology, approved all animal experiments (Permit Number: TJ2015A01). All surgeries were performed under sodium pentobarbital anesthesia, and all efforts were made to minimize animal suffering.

### Animals and Experimental Design

**Fig 1:**
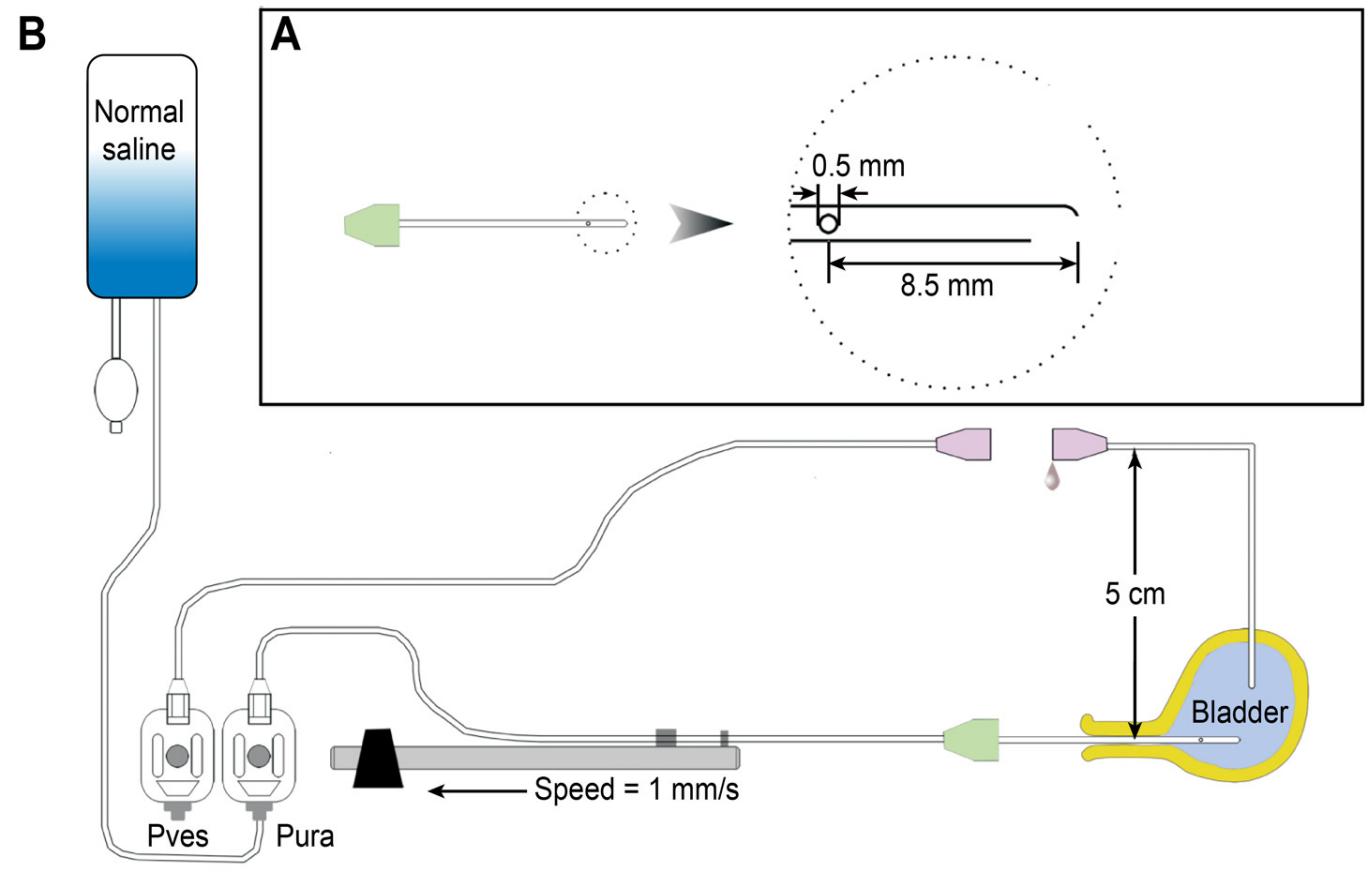
Schematic drawing of the setup for urethral pressure profile recording. (A) A lateral hole water-perfusion catheter, used for transurethral catheterization and UPP recording in female rats. (B) The general arrangement of the urodynamic device and the animal. Note that the suprapubic bladder catheter for the measuring of bladder pressure is disconnected and that both ends of this catheter are fixed at 5 cm above the 0 level (see text for details). *P*_*ves*_ = vesical pressure transducer; *P*_*ure*_ *=* urethral pressure transducer.

Seventeen female virgin Sprague-Dawley rats (8–12 weeks, 250–300 g) were used in this study. All animals were maintained in standard housing cages with free access to food and water and normal day/night cycling before the study. Sodium pentobarbital anesthesia (30 mg/kg intraperitoneally) was administered at study initiation. Each anesthetized animal was placed into the prone position on a foam plastic board using an elastic band and nails. A urodynamic device with a water-perfusion catheter system (Laborie, Toronto, Canada) was used for the whole-animal urodynamic study (Fig 1). Normal saline (Huayun shuanghe, Wuhan, China) was used for all intravesical and water-perfusion catheter infusions.

### Suprapubic Bladder Catheterization and LPP Determination

LPP was first determined using a suprapubic bladder tube in all of the female rats. A 15-cm-length 5 Fr. catheter (made from a ureteral catheter) was inserted into the bladder dome through an abdominal incision, and a purse-string suture was used to close the bladder dome incision tightly. The position of this catheter was adjusted so that only 0.5 cm was maintained in the bladder. The rest of the catheter was tunneled subcutaneously and secured with a hitch suture to the rectus fascia and lower abdominal skin to prevent slippage. The dimensions of this catheter and the transurethral water-perfusion catheter used for UPP recording were chosen based on the small body sizes of the female rats. Equal pressure transmission between the two catheters was demonstrated *in vitro* by elevating both ends of the transurethral and bladder catheters to a certain height after setting the pressure to 0 and *in vivo* by pressing the bladder body when both ends of the catheters were positioned in the bladder. The bladder catheter was connected to a pressure transducer within a urodynamic device via a tube with a 3-way connector. The bladder pressure value was digitized and recorded using urodynamic device software. Pressure activity was monitored on a computer screen, and the data were saved in the computer. The pressure transducer and pressure transmission tube system were examined to ensure that all air bubbles were eliminated, and the instrument was calibrated. Subsequently, 0.2 to 0.4 mL of room-temperature normal saline was infused slowly into the bladder. Stabilization of the initial bladder pressure at 15 ± 5 cm H_2_O was preferred prior to performing the LPP tests. The bladder body was pressed using a cotton stick, and urine leakage at the external meatus of the urethra was observed simultaneously, as previously described [22,23]. The bladder pressure at the time that leakage appeared at the meatus was recorded as the LPP. This process was repeated 3 to 4 times, and the average value was used for statistical analyses.

### Transurethral Catheterization and UPP Recording

UPP recordings were performed after LPP determinations. Rats have a relatively small bladder volume, and suprapubic bladder catheterization would further decrease this volume. Thus, saline infusion via a transurethral catheter during UPP recording would extend the bladder and increase bladder pressure, which could affect the UPP parameters. Therefore, we disconnected the suprapubic bladder and pressure transducer tubes to allow the fluid in the bladder to flow out freely. This procedure resulted in stabilization of the bladder volume and pressure during the UPP recordings and prevented the possible interference of variations in bladder volume and pressure on the UPP. The transurethral catheter opening and disconnected bladder catheter opening were set to the atmospheric pressure at the pubic symphysis level (i.e., set to 0), and calibration was performed. The end of the disconnected bladder pressure transducer tube and the outside opening of the suprapubic bladder catheter were fixed at 5 cm above the pubic symphysis to maintain the bladder pressure at 5 cm H_2_O throughout the entire UPP recording process.

A 5-cm 3 Fr. catheter made from an epidural anesthesia tube was prepared prior to the experiment (Fig 1B). These catheters generally have 3 fanning side holes near the end. We sealed 2 of the side holes with glue so that only one hole remained open. This catheter was connected to a pressure transducer via an adaptor and mounted to a mechanical withdrawal apparatus of the urodynamic device. The catheter was passed into the bladder via the urethra. Saline was infused via this transurethral catheter at 0.5 mL/min during UPP recording, and the catheter was simultaneously withdrawn at 1 mm/s by the mechanical withdrawal apparatus. Bladder pressure and urethral pressure values were digitized and recorded using urodynamic device software. The pressure readings were monitored on the computer screen, and the data were saved in the computer. The UPP recordings were performed with the lateral water-perfusion hole facing the 3-, 6-, 9-, and 12-o’clock positions in a randomized sequence in each rat, and four UPP curves were obtained at these 4 positions. These recordings were repeated in a randomized sequence to assess data reproducibility if the rat was performing well.

The rats were sacrificed at the end of this series of tests and autopsied to identify abnormalities in the urogenital system. Data from rats with grossly pathological organs were excluded from statistical analyses.

The general arrangement of the urodynamic device and the animal are presented in Fig 1 and S1 Fig.

### Statistical Analyses

The values for each parameter were averaged for each rat, and the results are reported as the mean ± standard deviation (SD). Data sets were tested for normality using the Kolmogorov-Smirnov (KS) test. Comparisons of the four positions of maximum urethral closure pressure (MUCP) among the data sets were performed using the Kruskal-Wallis test, followed by pair-wise multiple comparisons. Differences between the primary and repeated measurements of the MUCP and FUL were tested using paired-samples t tests (yielding two-tailed *p* values). The consistency of the UPP measurements between the primary and repeated recordings was assessed by calculating intra-class correlation coefficients (ICCs). In all cases, a *P* < 0.05 was considered statistically significant.

## Results

Seventeen adult female rats were prepared for the LPP test. Three of these rats exhibited unstable respiration during the procedure and ultimately died, and one of them exhibited a vaginal cervix mass at autopsy. The data from these 3 rats were excluded from analyses. The mean LPP of the remaining 14 rats was 64.39 ± 20.29 cm H_2_O. These 14 rats were used in the next step for UPP recording.

**Fig 2:**
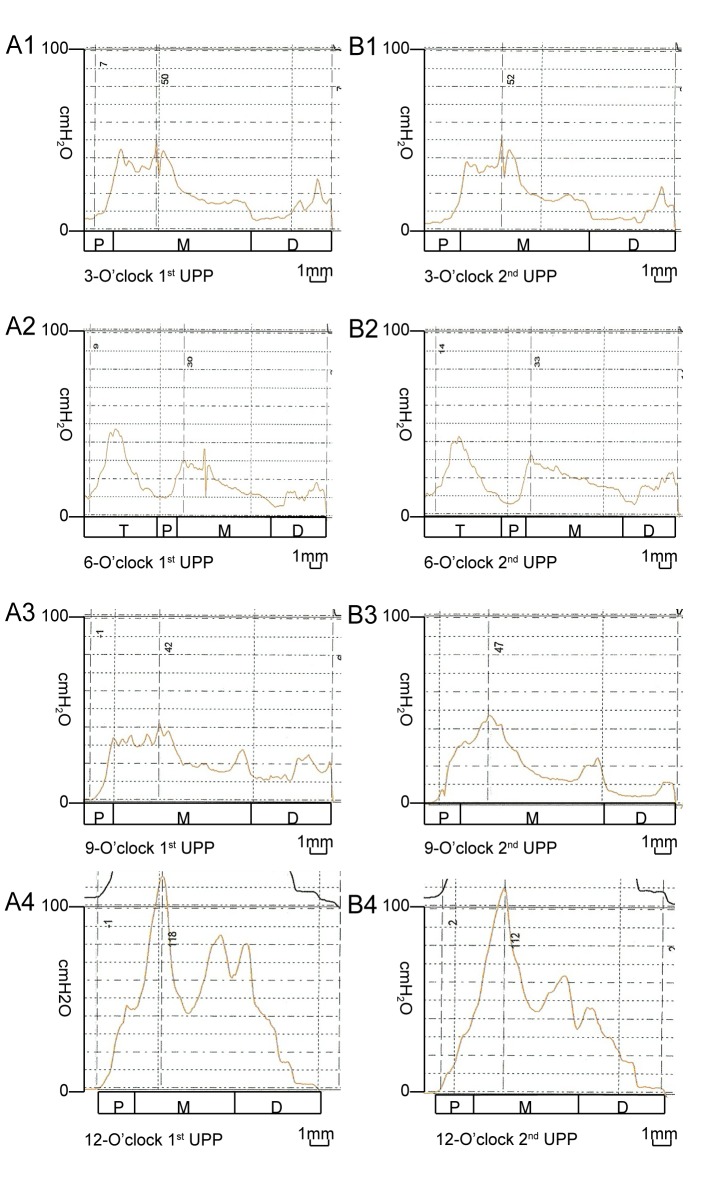
Example UPP recorded using a modified water-perfusion catheter system in 1 female rat. The left column (A1, A2, A3, and A4) shows the UPP curves, recorded primarily with the side holes oriented at the 3-, 6-, 9-, and 12-o’clock positions, respectively. The right column (B1, B2, B3, and B4) shows the repeated UPP curves, recorded with the side holes also oriented at the 3-, 6-, 9-, and 12-o’clock positions, respectively. Note the consistency between the primary and repeated UPP curves for the same position. The curves at different positions display remarkable variations in the patterns and pressure values. T: bladder trigone, P: proximal, M: mid, D: distal.

**Fig 3:**
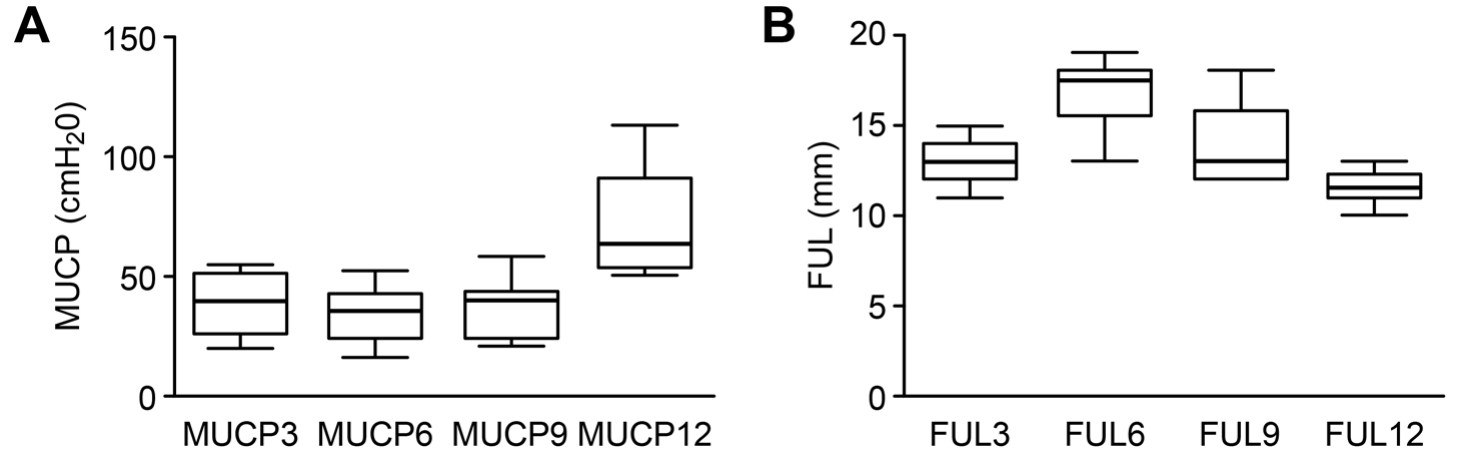
Comparisons of MUCP and FUL values at 4 different positions. (A) The MUCP values at 4 different positions. The bars represent the mean ± SD of the measurements. The highest MUCP value was observed at the 12-o’clock position compared with the other 3 positions (12- vs. 3-, *P* = 0.001; 12- vs. 6-, *P* < 0.001; 12- vs. 9-, *P* < 0.001). The MUCP values at the 3- and 9-o’clock positions are insignificantly different (3- vs. 9-, *P* = 0.702). (B) The FUL values at 4 different positions. The bars represent the mean ± SD. The longest FUL was observed at the 6-o’clock position compared with the other 3 positions (6- vs. 12-, *P <* 0.001; 6- vs. 3-, *P* = 0.005; 6- vs. 9-, *P* = 0.027). The FUL values at the 3- and 9-o’clock positions were similar (*P* = 0.540).

UPP recording was not completed in 4 of the 14 rats because of difficulties associated with transurethral catheterization. Therefore, UPP values from 10 rats were analyzed. Fig 2 shows an example of the UPP curves recorded in 1 female rat, including the primary and repeated recordings. The FUL values at the 3-, 6-, 9-, and 12-o’clock positions were 12.90 ± 1.20, 16.70 ± 1.95, 13.90 ± 2.42, and 11.60 ± 0.97 mm, respectively, and the MUCP values at these positions were 38.70 ± 11.85, 33.90 ± 11.82, 37.40 ± 11.95, and 71.90 ± 23.01 cm H_2_O, respectively. The highest MUCP value was observed at the 12-o’clock position compared with the other 3 positions (12- vs. 3-, *P* = 0.001; 12- vs. 6-, P < 0.001; 12- vs. 9-, P < 0.001), in addition to the lowest FUL value (12- vs. 6-, P < 0.001; 12- vs. 3-, P = 0.084; 12- vs. 9-, P = 0.019). The lowest MUCP value was observed at the 6-o’clock position (6- vs. 12-, *P <* 0.001; 6- vs. 3-, *P* = 0.473; 6- vs. 9-, *P* = 0.737), in addition to the highest FUL value (6- vs. 12-, *P <* 0.001; 6- vs. 3-, *P* = 0.005; 6- vs. 9-, *P* = 0.027). Similar MUCP (*P* = 0.702) and FUL (*P* = 0.540) values were detected at the 3- and 9-o’clock positions (Fig 3A and 3B). UPP recordings at all 4 positions were repeated in 9 rats. Table 1 shows the MUCP and FUL values obtained from the primary and repeated UPP recordings. There were no significant differences between these values, demonstrating the high reproducibility of the present method of UPP recording (*P* values are listed in Table 1). The ICC values for the MUCP and FUL are listed in Table 2. These values generally received high reliability scores. The ICC values for the MUCP between the primary and repeat recordings ranged from 0.838 to 0.960 with excellent reproducibility, and those for the FUL ranged from 0.672 to 0.844 with good reproducibility, further demonstrating the high reproducibility of this method of UPP recording.

**Table 1:** Comparisons of UPP parameters between the primary and repeatedrecordings (N = 9).

	Paired differences of MUCP			Paired differences of FUL		
Positions	Mean±SD	95% CI	*p*	Mean±SD	95% CI	*p*
3-o’clock primary-repeated	2.56±4.30	-0.75,5.86	0.113	-0.44±0.73	-1.00,0.11	0.104
6-o’clock primary-repeated	2.78±4.55	-0.72,6.27	0.104	-0.22±1.64	-1.48,1.04	0.695
9-o’clock primary-repeated	-0.11±5.97	-4.70,4.48	0.957	0.22±1.71	-1.10,1.54	0.708
12-o’clock primary-repeated	4.22±6.18	-0.53,8.97	0.075	-0.44±0.88	-1.12,0.23	0.169

The data are reported as the mean ± SD.

*P* values were calculated by the paired samples t-test.

MUCP: maximum urethral closure pressure

FUL: functional urethral length; 95%

CI: 95% confidence interval

SD: standard deviation.

**Table 2:** Consistency between the primary and repeated UPP recordings (N = 9).

	MUCP			FUL		
Positions	ICC	95% CI		ICC	95% CI	
3-o’clock	0.926	0.708	0.983	0.844	0.455	0.963
6-o’clock	0.916	0.675	0.980	0.672	0.070	0.915
9-o’clock	0.838	0.437	0.961	0.738	0.198	0.934
12-o’clock	0.960	0.835	0.991	0.720	0.162	0.929

The data are reported as the mean ± SD.

MUCP: maximum urethral closure pressure

FUL: functional urethral length

ICC: intra-class correlation coefficient; 95%

CI: 95% confidence interval.

## Discussion

We present a modified method for the recording of UPP in female rats. This method was developed on the basis of a water perfusion catheter system that was first described by Brown and Wickham. Our modifications utilize the most widely available urodynamic device and catheters.

Two modifications were made to adapt the use of this system in female rats because of their small body sizes. The first modification involved the transurethral catheter. Currently available transurethral catheters for human UPP recording have relatively larger dimensions and cannot be directly used in female rats [9,15–21,24–27]. We chose a 3 Fr. epidural anesthesia catheter with a single channel based on the small dimension of the rat urethra. Preliminary tests demonstrated that this catheter allowed for good water perfusion and pressure transmission. The second modification was made to overcome the limitation of the small volume of the bladder, which cannot accommodate the volume of water that typically flows in during a single UPP recording. An open suprapubic bladder catheter was used to resolve this problem by allowing the bladder water to flow out freely. The bladder pressure was fixed at approximately 5 cm H_2_O when we fixed the external end of the suprapubic bladder catheter and the end of the tube connected to the bladder pressure transducer at 5 cm above the pubic symphysis.

This method is simple and provides reliable and reproducible results. In this study, the entire length of the urethral wall, from the bladder neck to the external meatus, was evaluated. Unique UPP curves were recorded at 4 positions for each female rat. These preliminary results demonstrated that the UPP curves recorded using this method sensitively and appreciably reflected the different pressures exerted by the urethral wall. These female rats exhibited a relatively consistent UPP pattern, which was highly reproducible, and each urethra exhibited a characteristic pressure profile. The whole UPP curve can be divided into 3 segments. The first segment revealed a definite progressive increase in pressures, which represented the values at recording sites in the proximal urethral segment. The pressures in the second segment peaked and maintained a high level for a certain length, which represented the values at recording sites in the mid-urethral segment. The pressures in the third segment progressively dropped and ended at approximately the 0 level, which represented the distal urethral segment. The anatomical localizations of points of high and low pressures were easily performed using catheter calibration. Therefore, this method provides a useful tool for the investigation of the anatomy and physiology of urethra in this small animal species.

The UPP curves in female rats bear many similarities to those of women. Similar patterns of UPP curves and pressure levels of MUCP were reported previously in human females [16,17]. The most intriguing result is that the positional differences demonstrated UPP recordings in women were also demonstrated in female rat UPP recordings [16,17]. Only a few papers in the literature discuss the directional differences of UPP in human females, and two contrary opinions exist [16,17]. Some groups consider these differences to be artifacts. A hypothesis has been proposed that the passage of a flexible but straight catheter through a curved urethra results in application of additional forces on the transducer caused by catheter bending [15,28]. Other groups consider these differences to be physiological phenomena. An active perineal contraction may explain the anisotropic rotational pressure variations in the urethra [14,29]. Our observations revealed new facts about these positional differences. The patterns of UPPs and their parameters between the left and right halves (i.e., 3 o’clock and 9 o’clock, respectively) were symmetrical. These observations suggest that positional differences in UPP should not be oversimplified as artifacts. These specific features of pressure distribution in the female rat urethra and their relationships with urethral anatomical structures and physiological status will be addressed in future studies.

In human urodynamic studies, LPP and UPP measurements as well as EUS-EMG and videourodynamic analyses are commonly performed to examine urethral function or pathophysiology. LPP and UPP measurements are quantitative, while EUS-EMG and videourodynamic analyses are semi-quantitative. In clinical practice/basic science, various combinations of these methods are needed, depending on the diagnosis/scientific purpose. Most of these methods, with the exception of UPP measurement, have been widely used on small-sized animals, such as rats. LPP measurements and EMG have been successfully performed in anesthetized and fully awake rats [1,10,11]. Our methodology provides a simple technique for performing UPP recordings in anesthetized rats. With further modifications, there is possibility of incorporating UPP in fully awake rats urodynamic study, which will develop an assessment protocol in rat models in close analogy to the urodynamic assessment used clinically in humans. The development of this protocol will further enhance the valve of rat model in the study of lower urinary tract dysfunction.

### Study Limitations

The present study has several limitations as a primary methodological investigation. First, the suprapubic bladder catheter was open to the air to overcome the limitation of the small bladder volumes of female rats, which obviously does not represent the physiological situation. The physiological pressure level was approached by lifting the suprapubic bladder catheter opening to a height of 5 cm H_2_O in our study. However, the stress UPP was still not able to be determined. The stress UPP in female rats may be determined by lifting the suprapubic bladder catheter opening to a different height range (e.g., 30 cm H_2_O or more) or connecting an elastic bag to enlarge the bladder volume. Further investigations should be conducted to standardize UPP and stress UPP recordings.

Second, we used sodium pentobarbital for anesthesia because this anesthetic is the most widely used in our laboratory, and it provides satisfactory anesthesia to allow for completion of UPP recordings. However, different anesthetics may have different effects on urodynamic parameters, such as cystometry and LPP [30–32]. The present study focused on the modification of the UPP recording technique, and we did not investigate whether different anesthetics differentially affected UPP recording. Future studies comparing different anesthetics on UPP recording outcomes should be performed to address this issue.

Finally, a full understanding of how each unique UPP curve is formed and what factors affect the UPP parameters is essential to a better understanding of the physiological relevance and clinical implications of these recordings. The present study did not address this complex issue. Elucidation of the corresponding relationships between UPP curve patterns and the anatomical and histological structures in the female rat urethra will be discussed in subsequent reports.

## Conclusions

UPP recording using a modified method based on a water-perfusion catheter system is feasible and reproducible in female rats. This study is the first report of UPP recordings in female rats that are similar to those that have been reported in women. These results will likely enhance the utility of the female rat model for *in vivo* investigations of urethral function and of the mechanisms of urinary continence in humans.

## Supporting Information

S1 Fig The use of a modified water-perfusion catheter system with a urodynamic device for the recording of UPP in female rats. (A) The general arrangement of the urodynamic device and animal. The pressure transducer and rat pubic symphysis were set at same level. The tube end of the pressure transducer and the side hole of the water-perfusion catheter were always set to 0 at the pubic symphysis. (B) The water-perfusion catheter with 1 side hole for transurethral catheterization and recording the urethral pressure. (C) The water-perfusion catheter was inserted into bladder transurethrally and later mounted to the mechanical withdrawer. (D) The suprapubic bladder catheter and the pressure transducer tube were disconnected, and both ends were opened to air. The ends were fixed 5 cm above the 0 level. Therefore, the water perfusing into the bladder during UPP recordings flowed out freely to avoid overextension of the bladder. The bladder pressure was fixed at 5 cm H_2_O (see text for details).
(TIF)
