# Supplementary figures and images for: A Method for Recording Urethral Pressure Profiles in Female Rats
Source: PLoS One. 2015 Oct 26;10(10):e0140851. doi: 10.1371/journal.pone.0140851 (PMC4621020; doi:10.1371/journal.pone.0140851)

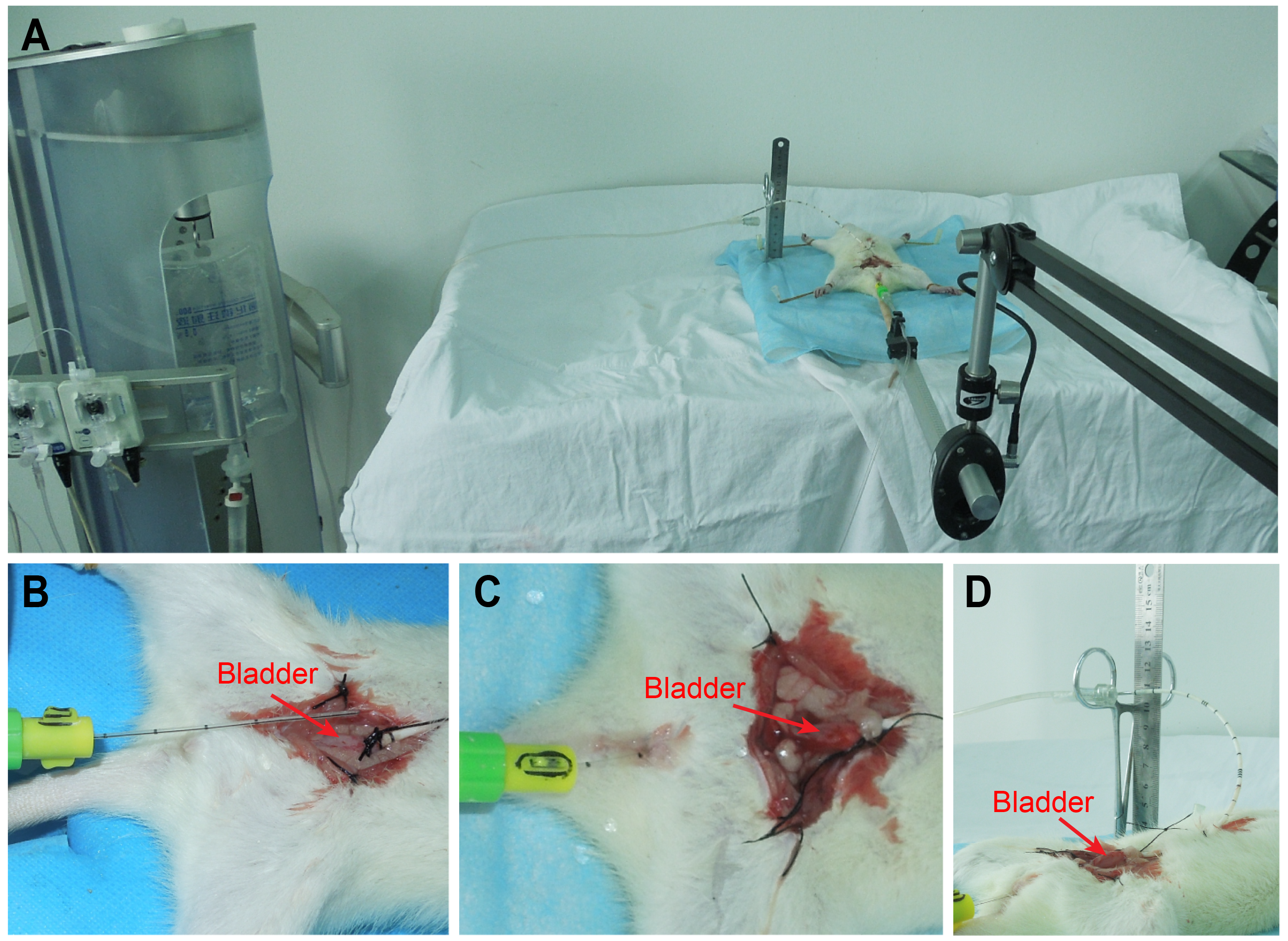

Supplement: S1 Fig — (A) The general arrangement of the urodynamic device and animal. The pressure transducer and rat pubic symphysis were set at same level. The tube end of the pressure transducer and the side hole of the water-perfusion catheter were always set to 0 at the pubic symphysis. (B) The water-perfusion catheter with 1 side hole for transurethral catheterization and recording the urethral pressure. (C) The water-perfusion catheter was inserted into bladder transurethrally and later mounted to the mechanical withdrawer. (D) The suprapubic bladder catheter and the pressure transducer tube were disconnected, and both ends were opened to air. The ends were fixed 5 cm above the 0 level. Therefore, the water perfusing into the bladder during UPP recordings flowed out freely to avoid overextension of the bladder. The bladder pressure was fixed at 5 cm H2O (see text for details). (TIF) [file pone.0140851.s001.tif]
